# 0.5 V, nW-Range Universal Filter Based on Multiple-Input Transconductor for Biosignals Processing

**DOI:** 10.3390/s22228619

**Published:** 2022-11-08

**Authors:** Fabian Khateb, Montree Kumngern, Tomasz Kulej, Meysam Akbari, Viera Stopjakova

**Affiliations:** 1 Department of Microelectronics, Brno University of Technology, Technická 10, 601 90 Brno, Czech Republic; 2 Faculty of Biomedical Engineering, Czech Technical University in Prague, nám. Sítná 3105, 272 01 Kladno, Czech Republic; 3 Department of Electrical Engineering, University of Defence, Kounicova 65, 662 10 Brno, Czech Republic; 4 Department of Telecommunications Engineering, School of Engineering, King Mongkut’s Institute of Technology Ladkrabang, Bangkok 10520, Thailand; 5 Department of Electrical Engineering, Czestochowa University of Technology, 42-201 Czestochowa, Poland; 6 Department of Electrical Engineering, University of Kurdistan, Sanandaj 66177-15175, Iran; 7 Faculty of Electrical Engineering and Information Technology, Slovak University of Technology, 81219 Bratislava, Slovakia

**Keywords:** OTA, multiple-input MOS transistor, low-voltage low-power, universal filter, biosignals processing

## Abstract

This paper demonstrates the advantages of the multiple-input transconductor (MI-G_m_) in filter application, in terms of topology simplification, increasing filter functions, and minimizing the count of needed active blocks and their consumed power. Further, the filter enjoys high input impedance, uses three MI-G_m_s and two grounded capacitors, and it offers both inverting and non-inverting versions of low-pass (LPF), high-pass (HPF), band-pass (BPF), band-stop (BS) and all-pass (AP) functions. The filter operates under a supply voltage of 0.5 V and consumes 37 nW, hence it is suitable for extremely low-voltage low-power applications like biosignals processing. The circuit was designed in a Cadence environment using 180 nm CMOS technology from Taiwan Semiconductor Manufacturing Company (TSMC). The post-layout simulation results, including Monte Carlo and process, voltage, temperature (PVT) corners for the proposed filter correlate well with the theoretical results that confirm attractive features of the developed filter based on MI-G_m_.

## 1. Introduction

**Figure 1:**
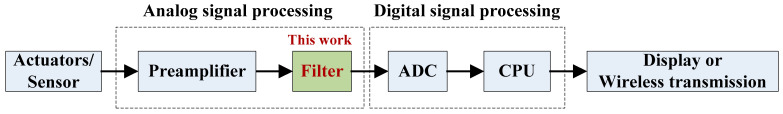
The conceptual diagram for processing biosignals.

The innovations in circuit design techniques for low-voltage supply and low-power consumption for portable electronics, energy harvesting, biomedical monitoring, and autonomous sensor applications are vital [1,2,3,4]. For biosignal processing electronics, where the bio-signals spectrum lies between sub-hertz up to 10 kHz, the extremely low-voltage supply and low-power consumption of such electronics are rather beneficial since it prolongs the operating lifetime of these applications. Figure 1 shows a conceptual diagram of biosignals processing, where the biosignals with very low amplitude (in the range from µV up to mV) are sensed by actuators/sensors. Then, the sensed signals are amplified by a low-noise amplifier (preamplifier), and the unwanted noise is removed by a suitable analog filter, which is the target of this paper. Next, the digital signal processing includes an analog-to-digital converter (ADC) and a central processing unit (CPU). The resulting data are displayed or wirelessly transmitted.

**Figure 2:**
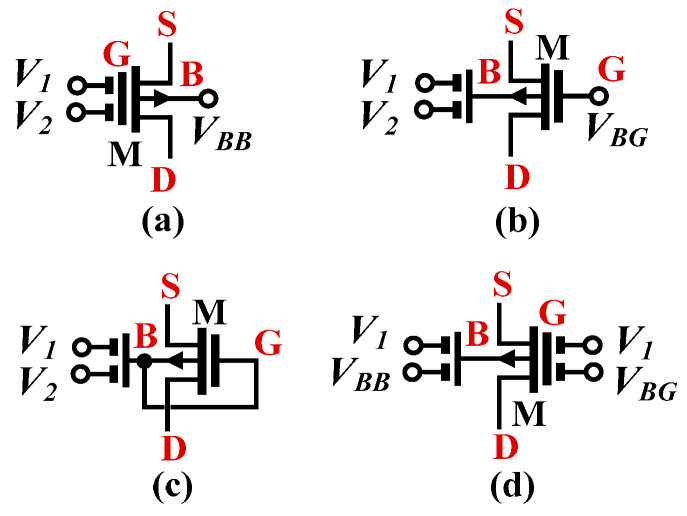
Symbol of the multiple-input MOS transistor: gate (**a**), bulk (**b**), DTMOS (**c**) and QFG (**d**).

The operational transconductance amplifier (OTA), also known as the transconductor (*G_m_* stage), is a basic block for electronic applications like filters and oscillators [5,6,7,8,9,10]. Unlike the standard and well-known single-input OTA, the multiple-input OTA/transconductor (MI-OTA/MI-G_m_) offers increased arithmetic operation at the input that results in a reduced number of active elements, power consumption, and simplification of the filter topology. It is worth noting that for designers in CMOS, it is a challenge to design a circuit operating with supply voltage V_DD_ around or even below the threshold voltage V_TH_ of the MOS transistor without scarifying the performance of the circuit. The use of multiple-input transconductors to reduce the number of components in the design of OTA-C filters was confirmed in the literature [5,6]. It was shown that the multiple-input OTA can reduce the number of components, silicon area, and power dissipation by approximately factor k, where k is the number of OTA inputs [5]. Multiple-input transconductor can be obtained by the following techniques: 1. using extra differential pairs [5,6], or 2. using a multiple-input floating-gate transistor (MIFG) [7,8,9,10]. While the first technique increases the count of transistors, current branches, and the complexity of the design, the second technique suffers from the high-voltage offset, incapability of processing DC signals, and becomes unsuitable for modern deep-nanoscale CMOS technology with gate leakage [11]. A promising technique that offers multiple-input OTA without the above-mentioned limitations is the multiple-input MOS transistor (MI-MOS), firstly presented and experimentally confirmed in [12,13,14]. The multiple-input MOS transistor is shown in Figure 2. The multiple-input terminals V_1_, V_2_, etc. can be obtained from: a. the gate while the bulk is biased by voltage V_BB_, b. from the bulk while the gate is biased by V_BG_, c. from the bulk-gate (known as dynamic threshold MOS transistor “DTMOS”) without biasing or d. from the bulk-gate (known as quasi-floating-gate “QFG”) with different biasing voltages V_BB_ and V_BG_ for bulk and gate, respectively [15].

**Figure 3:**
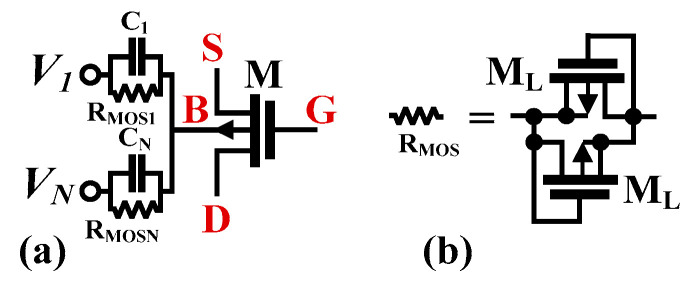
MI-BD MOS transistor (**a**), and realization of R_MOS_ (**b**).

The realization of the multiple-input with bulk-driven MOS device is shown in Figure 3. The multiple-input is constructed by a capacitive summing circuit using capacitors *C_i_* (*i* = 1,…,N) connected to the bulk terminal of a MOS transistor. To provide proper biasing of the bulk terminal for DC operation, the high resistance resistors *R_MOS_* is used. These *R_MOS_* are realized as the anti-parallel connection of two minimum-size transistors M_L_, operating with *V_GS_* = 0. For AC signals, and for frequencies *f* >> 1/2π*C_i_R_MOSi_*, *i* = 1…N, resistors *R_MOS_* are shunted by capacitances *C_i_*, which create an analog voltage divider/voltage summing circuit, with the gain coefficients determined solely by the ratio of capacitances [15].

In this work, the multiple-input bulk-driven MOS transistor is implemented using a CMOS structure of the *G_m_* to build a multiple-input voltage-mode analog filter. As a result, the number of used active devices is reduced while offering more filtering responses compared to conventional G_m_-based filters. 

## 2. Methods

In this section, the design of the multiple-input *G_m_* and the universal filter based on it will be described. 

### 2.1. The Multiple-Input G_m_

**Figure 4:**
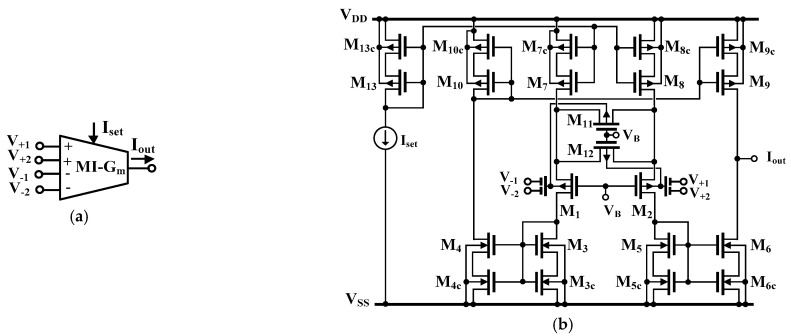
The symbol of the multiple-input *G_m_* stage (**a**) and its CMOS structure (**b**).

The symbol and CMOS structure of the MI-G_m_ stage are shown in Figure 4a,b, respectively. In an ideal case, the transfer characteristic of the MI-G_m_ stage of Figure 4a can be expressed by:

(1) $$I_{out}=G_{m}\left(V_{+1}+V_{+2}-V_{-1}-V_{-2}\right),$$

where *G_m_* is the transconductance gain, *V_+_*_1_ and *V_+_*_2_ are signals at the non-inverting inputs, *V_−_*_1_, *V_−_*_2_ are signals at the inverting inputs, and *I_out_* is the output current. 

The particular realization of the MI-G_m_ stage discussed here was first presented and experimentally verified in [15]. The circuit employs the MI-bulk-driven differential pair M_1_, M_2_, with the source-degenerative bulk-driven transistors M_11_, M_12_, which operate in the triode region and improve the circuit linearity. Note, that *V_GS_* as well as *V_BS_* voltages for M_11_, M_12_ and M_1_, M_2_ are identical for any common-mode input voltage and biasing current. The single-input gate-driven counterpart of the input stage was first proposed in [16], and its weak-inversion version was discussed in [17]. Here, due to the use of bulk-driven transistors, and an additional capacitive voltage divider, both, the input linear range, as well as the input common-mode range are significantly increased, as compared with the conventional gate-driven (GD) version operating in a weak-inversion region. Moreover, the application of MI transistors allows realizing MI-G_m_s without multiplying the input differential pair, as in classical solutions, which saves power and simplifies the overall structure of such circuits.

Regarding the rest of the structure, the circuit can be seen as a classical current-mirror OTA, where all current mirrors are realized with the use of self-cascode transistors. This improves their output resistances, and consequently, also the DC voltage gain of the proposed OTA, with negligible limitation of the output voltage swing. Note, that the current gain of all current mirrors in this design was assumed to be equal to unity.

Assuming that a p-MOS transistor is operating in a weak-inversion region, the drain current can be described by the following equation, [18]:

(2) $$I_{D}=I_{T}\left(\frac{W}{L}\right)exp\left(\frac{V_{SG}+V_{TH}}{n_{p}U_{T}}\right)\left[1-exp\left(-\frac{V_{SD}}{U_{T}}\right)\right]$$

where *I_T_* is the technology current, *W* and *L* are the transistor channel width and length, respectively, *n_p_* is the subthreshold slope factor, *U_T_* is the thermal potential and *V_TH_* is the threshold voltage, which can be linearly approximated as:

(3) $$V_{TH}=V_{TO}-\left(n_{p}-1\right)V_{BS\;\;}$$

where *V_TO_* is the threshold voltage for *V_BS_* = 0.

Assuming that the circuit is controlled with *i*-th differential input, with other inputs grounded for AC signals, the low-frequency large-signal transfer characteristic of the *G_m_* can be expressed as:

(4) $$I_{out}=2I_{set}tanh\left(\beta_{i}\eta \frac{V_{+i}-V_{-i}}{2n_{p}U_{T}}-tanh^{-1}\left[\frac{1}{4m+1}tanh\left(\beta_{i}\eta \frac{V_{V_{+i}-V_{-i}}}{2n_{p}U_{T}}\right)\right]\right)$$

where *η* = (*n_p_* − 1)=*g_mb_*_1,2_/*g_m_*_1,2_ at the operating point, *m*= (*W*_11_/*L*_12_)⁄(*W*_1_/*L*_1_) is the relative aspect ratio of the two matched transistor pairs M_11_–M_12_ and M_1_–M_2._ *β_i_* is the voltage gain of the input capacitive divider from one input, which neglects the second order effects and for *f* >> 1/*C_i_R_MOSi_* can be approximated as:

(5) $$\beta_{i}≅\frac{C_{i}}{\sum_{i=1}^{n}C_{i}}$$

where *n* is the total number of differential inputs (in the discussed design *n* = 2).

For optimum linearity, the coefficient m should be equal to 0.5, as for the GD counterpart, of the discussed circuit. This value does not depend on the biasing voltage *I_set_* [17].

As it can be concluded from Equation (4), as compared to its single-input GD counterpart, the linear range of the proposed circuit is extended by a factor of 1⁄*β_i_η*, which for the discussed case (*β_i_* = 0.5, *η* = 0.34) means that the linear range is extended around 6 times. 

The small-signal transconductance of the *G_m_* can be calculated from Equation (4) as:

(6) $$G_{m}=\beta_{i}\eta \cdot \frac{4m}{4m+1}\cdot \frac{I_{set}}{n_{p}U_{T}}$$

thus, the small-signal transconductance is equal to the gate transconductance of the input transistors M_1_ and M_2_, multiplied by a factor of [4*m*⁄(4*m* + 1)] *β_i_η*, which for the proposed design in the optimal case (*m* = 0.5) is equal to around 1⁄9.

The low-frequency voltage gain of the *G_m_* can be approximated as:

(7) $$A_{VO}≅G_{m}[\left(g_{m9}r_{dsD9}r_{ds9c}\right)||\left(g_{m6}r_{ds6}r_{ds6c}\right)]$$

Its value is negatively affected by the low transconductance of the MI-G_m_. On the other hand, however, self-cascode connections allow for enlarging the output resistance of the MI-G_m_, thus improving its voltage gain and at least partially compensating the losses caused by the input capacitive divider and the small bulk transconductance of MOS transistors.

Assuming that the noise current of an *i*-th MOS transistor in a weak inversion region can be expressed as:

(8) $$\bar{I_{ni}^{2}}=2qI_{Di}+\frac{1}{fC_{OX}}\left(\frac{Kg_{mi}^{2}}{W_{i}L_{i}}\right)$$

where *q* is the electron charge, *C_OX_* is the oxide capacitance per unit area and *K* is the flicker noise constant, the input-referred noise of the MI-G_m_, referred to as one of the differential inputs, is given by:

(9) $$\bar{v_{n}^{2}}=\frac{1}{G_{m}^{2}}\left[2\bar{I_{n1,2}^{2}}{\left(\frac{2G}{g_{m1,2}+2G}\right)}^{2}+2\bar{I_{n7,8SC}^{2}}{\left(\frac{g_{m1,2}}{g_{m1,2}+2G}\right)}^{2}\;++\bar{I_{nG}^{2}}{\left(2\frac{g_{m1,2}}{g_{m1,2}+2G}\right)}^{2}+4\bar{I_{n3-6SC}^{2}}+2\bar{I_{n9,10SC}^{2}}\right]$$

where *G* = 1⁄(*r_ds_*_11_||*r_ds_*_12_) at the operating point.

As it can be concluded from Equation (9), the input-referred noise of the MI-G_m_ is increased, as compared to its single-input GD counterpart, due to the lower transconductance *G_m_*. However, the input noise is increased in the same proportion as the input linear range, therefore, the dynamic range will not be affected and remains the same in both realizations.

### 2.2. Universal Filter Design 

The voltage-mode analog filter is a commonly used analog signal processing block, that is well-known for a long time. This is due to the versatility of operational amplifiers that are commonly used in the synthesis of analog electronic circuits [19]. Over the last decades, some other active elements such as operational transconductance amplifiers (OTAs), second-generation current conveyors (CCIIs), and current feedback operational amplifiers (CFOAs) have received considerable attention for designing voltage- and current-mode analog filters [20,21,22,23,24,25,26,27,28]. To design voltage-mode filters, multiple-input type filters can reduce the number of active devices compared with single-input type filters, because variant filtering responses can be obtained by appropriately applying the input signal, depending on the conditions of the required filtering responses. To avoid loading effects, the input terminals of the voltage-mode filter must have high impedance. To avoid additional circuits such as inverting amplifiers, the minus-type input signal of voltage-mode filters must be available.

**Figure 5:**
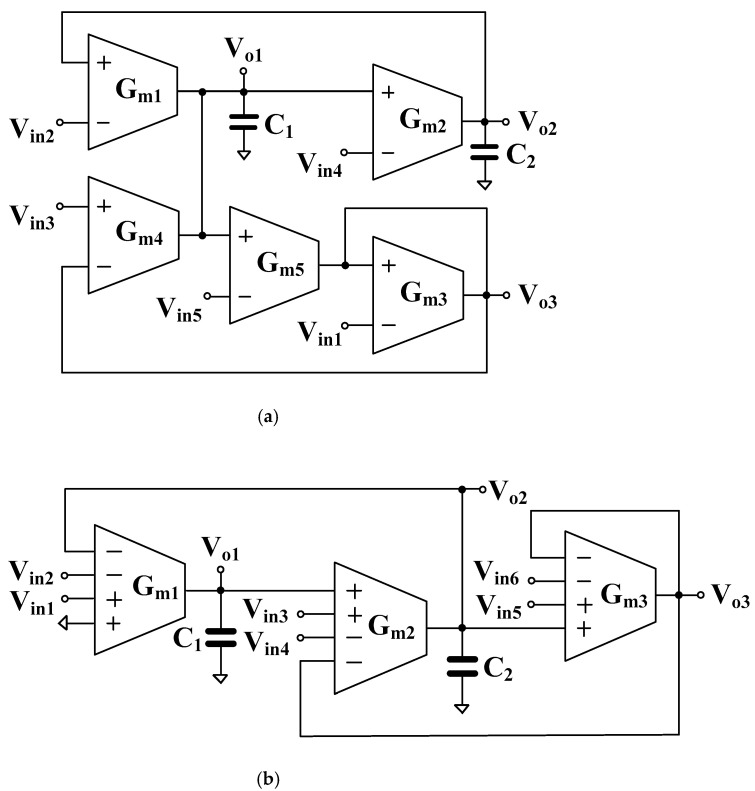
Proposed universal filter using standard *G_m_* [26] (**a**), and MI-G_m_ (**b**).

For the purpose of illustration, Figure 5a shows a universal filter design using five standard *G_m_* blocks, and two grounded capacitors and it offers five standard filtering functions [26]. In this work, a multiple-input voltage-mode analog filter using multiple-input transconductors MI-G_m_ is proposed as shown in Figure 5b. The structure will show that the multiple-input G_m_-based filter can reduce the number of used active devices and can offer more filtering responses compared with conventional G_m_-based filters. The filter employs three multiple-input *G_m_* stages and two grounded capacitors, which is desirable in integrated solutions. Thanks to the MI-G_m_ elements that offer noninverting/inverting multiple-input terminals, noninverting/inverting transfer functions of five types of filtering responses, namely, low-pass, high-pass, band-pass, band-stop, and all-pass can be easily obtained. Moreover, the input signals are connected to the high-impedance inputs of MI-G_m_, hence the additional buffer circuits to avoid the loading effects are not required. It is worth noting that although both filters in Figure 5a,b offer the five standard filtering functions, the count of active elements is reduced from 5 to 3 thanks to the MI-G_m_. This results in power consumption reduction and filter topology simplification, and in offering more transfer functions (including both non-inverting and inverting versions of five standard filtering functions).

Using Equation (1) and nodal analysis, the output voltages of Figure 5b are given by

(10) $$V_{o1}=\frac{\left(sC_{2}G_{m1}+G_{m1}G_{m2}\right)\left(V_{in1}-V_{in2}\right)+G_{m1}G_{m2}\left(V_{in4}-V_{in3}+V_{in5}-V_{in6}\right)}{s^{2}C_{1}C_{2}+sC_{1}G_{m2}+G_{m1}G_{m2}}$$

(11) $$V_{o2}=\frac{G_{m1}G_{m2}\left(V_{in1}-V_{in2}\right)+C_{1}G_{m2}\left(V_{in3}-V_{in4}+V_{in6}-V_{in5}\right)}{s^{2}C_{1}C_{2}+sC_{1}G_{m2}+G_{m1}G_{m2}}$$

(12) $$V_{o3}=\frac{G_{m1}G_{m2}\left(V_{in1}-V_{in2}\right)+C_{1}G_{m2}\left(V_{in3}-V_{in4}\right)+(s^{2}C_{2}C_{2}+G_{m1}G_{m2})\left(V_{in5}-V_{in6}\right)}{s^{2}C_{1}C_{2}+sC_{1}G_{m2}+G_{m1}G_{m2}}$$

The conditions for obtaining variant filtering responses by the appropriate connection of input signals are shown in Table 1.

The natural frequency (*ω_o_*) and the quality factor (*Q*) are given by:

(13) $$\omega_{o}=\sqrt{\frac{G_{m1}G_{m2}}{C_{1}C_{2}}}$$

(14) $$Q=\sqrt{\frac{C_{2}G_{m1}}{C_{1}G_{m2}}}$$

It is apparent that the parameter *ω_o_* can be controlled electronically by *G_m_*_1_ = *G_m_*_2_ while the parameter *Q* is controllable orthogonally by the ratio of *C*_2_/*C*_1_.

Taking into account the non-idealities of MI-G_m_, there are three major non-idealities that should be considered [29]: (i) the frequency-dependent transconductance, (ii) the input parasitic resistances and capacitances, (iii) the output parasitic resistances and capacitances.

**Figure 6:**
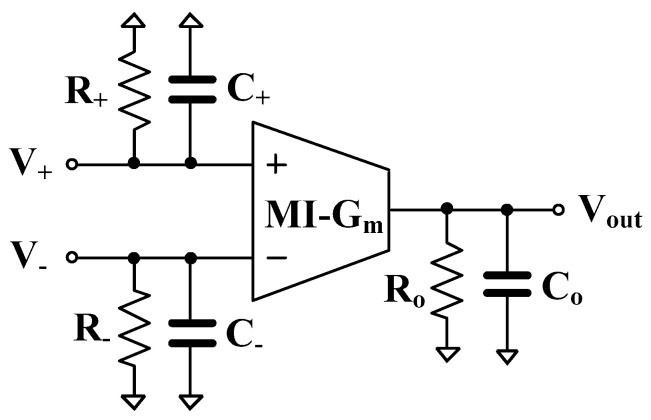
Non-ideal MI-G_m_ model with parasitic elements.

Figure 6 shows the non-ideal model with parasitic elements of the MI-G_m_, where *R_+_*, *R_−_*, *C_+_*, *C_−_* are the input parasitic resistances and capacitances, and *R_o_*, *C_o_* is the output parasitic resistance and capacitance, respectively. Considering Figure 5b the parasitic resistances at nodes *V_o_*_1_ and *V_o_*_2_ are, respectively, *R_o_*_1_//*R_+_*_1_ and *R_o_*_2_//*R_+_*_3_, thus the value of these parallel resistances is very high and can be neglected. Consider the parasitic capacitances at nodes *V_o_*_1_ and *V_o_*_2_, they can be expressed respectively as $C_{1}^{\prime }=C_{1}+C_{o1}+C_{+2}$ and $C_{2}^{\prime }=C_{2}+C_{o2}+C_{-1}+C_{+3}$.

Considering the non-ideality of transconductance, the output current can be rewritten as

(15) $$I_{out}=G_{mnj}\left(V_{+1}+V_{+2}-V_{-1}-V_{-2}\right),$$

where *G_mnj_* is the non-ideal transconductance gain of the *j*-th MI-G_m_ that is frequency-dependent, and can be approximately given by [29,30]:

(16) $$G_{mnj}\left(s\right)≅G_{mj}\left(1-T_{j}s\right)$$

From Figure 5 and Equation (16), denominators of Equations (10)−(12) can be expressed by:

(17) $$s^{2}C_{1}^{\prime }C_{2}^{\prime }\left(1-\frac{C_{1}G_{m2}T_{2}-G_{m1}G_{m2}T_{1}T_{2}}{C_{1}C_{2}}\right)+sC_{1}^{\prime }G_{m2}\left(1-\frac{G_{m1}G_{m2}T_{1}+G_{m1}G_{m2}T_{2}}{C_{1}^{\prime }G_{m2}}\right)\;+G_{m1}G_{m2}$$

The non-idealities of the transconductance $G_{mnj}$ can be neglected, if the following condition is satisfied:

(18) $$\frac{C_{1}^{\prime }G_{m2}T_{2}-G_{m1}G_{m2}T_{1}T_{2}}{C_{1}^{\prime }C_{2}^{\prime }}\ll 1$$

(19) $$\frac{G_{m1}G_{m2}T_{1}+G_{m1}G_{m2}T_{2}}{C_{1}^{\prime }G_{m2}}\ll 1$$

In such a case, the parameters *ω_o_* and *Q* become as follows:

(20) $$\omega_{o}=\sqrt{\frac{G_{m1}G_{m2}}{C_{1}^{\prime }C_{2}^{\prime }}}$$

(21) $$Q=\sqrt{\frac{C_{2}^{\prime }G_{m1}}{C_{1}^{\prime }G_{m2}}}$$

The parasitic capacitances will decrease the value of *ω_o_* as compared to the ideal case.

## 3. Results and Discussion 

**Figure 7:**
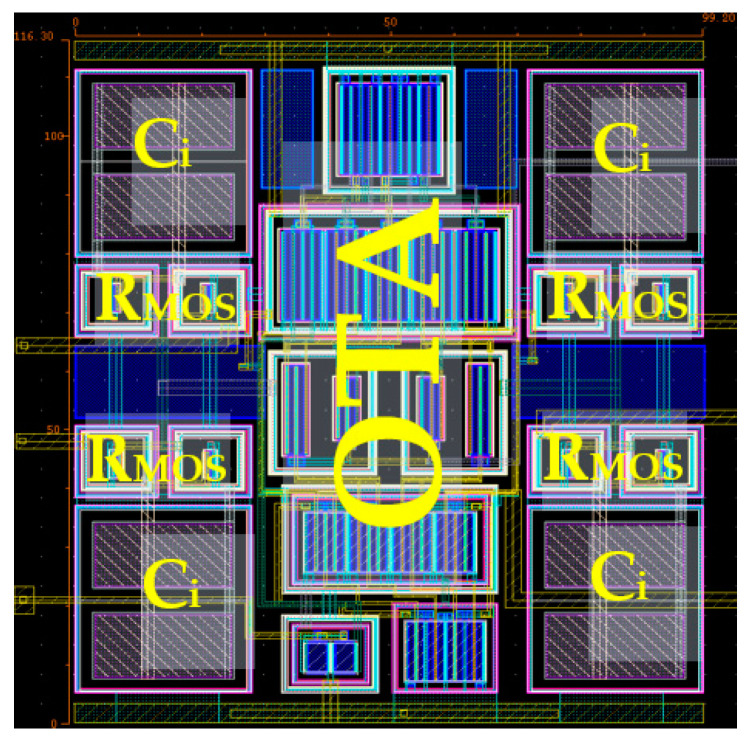
The layout of the MI-G_m_.

The filter circuit was designed in a Cadence environment using 180 nm TSMC CMOS technology. The voltage supply was 0.5 V, and the power consumption of the filter was 37 nW. The MI-G_m_ stage first presented in [15] was used. The transistor aspect ratios W/L are presented in Table 2. The input metal-insulator-metal (MIM) capacitor *C_i_* with a capacitance value of 0.5 pF was used. The layout of the MI-G_m_ is shown in Figure 7, with a silicon area of 116.3 µm × 99.2 µm.

**Figure 8:**
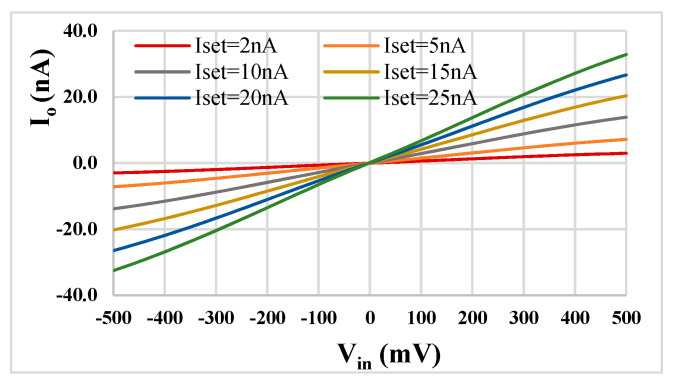
DC transfer characteristic of the MI-G_m_.

The DC transfer characteristics of the used MI-G_m_ for *I_set_* = [2, 5, 10, 15, 20, 25] nA are shown in Figure 8. The enhanced linearity in the *V_in_* range of ±500 mV is clearly observable.

**Figure 9:**
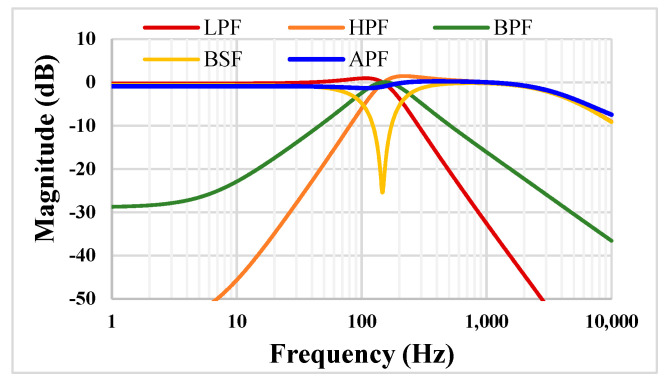
The simulated frequency responses of the proposed filter.

For the filter application, the simulated frequency responses of the proposed filter are shown in Figure 9. The values of *C*_1_ = *C*_2_ = 15 pF and the setting current *I_set_* = 5 nA. The simulated cut-off frequency value of 153 Hz is very close to the calculated value of 154.9 Hz. The power consumption of the filter was 37 nW.

**Figure 10:**
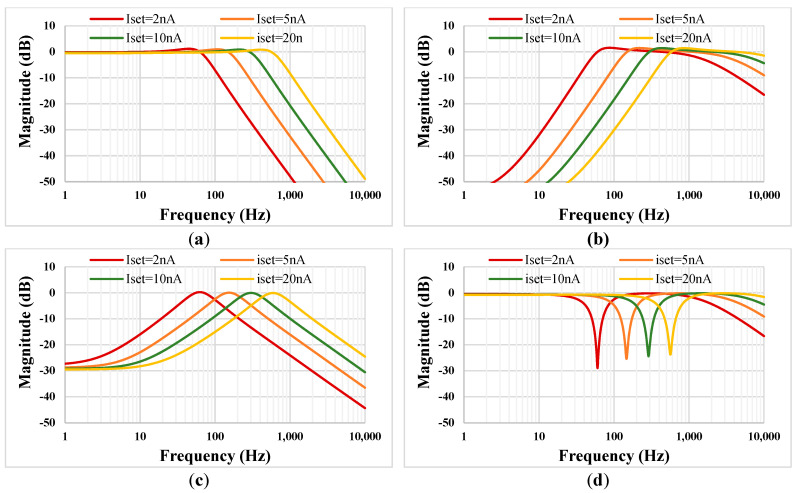
The simulated tuning capability of the proposed LPF (**a**), HPF (**b**), BPF (**c**) and BSF (**d**).

Figure 10 shows the tuning capability of the LPF (a), HPF (b), BPF (c), and BSF (d) with *C*_1_ = *C*_2_ = 15 pF. The setting current was *I_set_* = 2 nA, 5 nA, 10nA, and 20 nA and the cut-off frequency values were 62.3 Hz, 153 Hz, 301.9 Hz, and 595.6 Hz, respectively. Results shown in Figure 10 confirm the wide tuning capability of the proposed filter for low-frequency biomedical applications.

**Figure 11:**
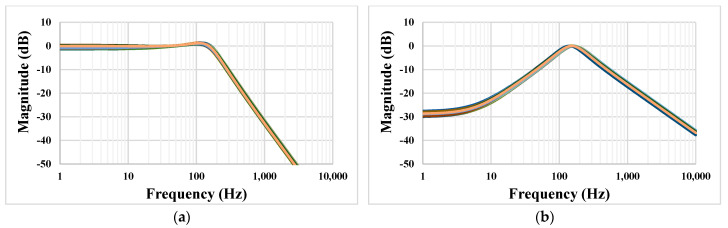
The Monte Carlo simulation of the LPF (**a**) and BPF (**b**).

The Monte Carlo process and mismatch analysis was performed with 200 runs. Figure 11 shows the simulated results for the LPF and BPF. The low-frequency gain at 1 Hz of the LPF was in the range from −1.39 dB to 0.47 dB, and the gain of the BPF at a frequency of 153 Hz was in the range from −0.438 dB to 0.168 dB.

**Figure 12:**
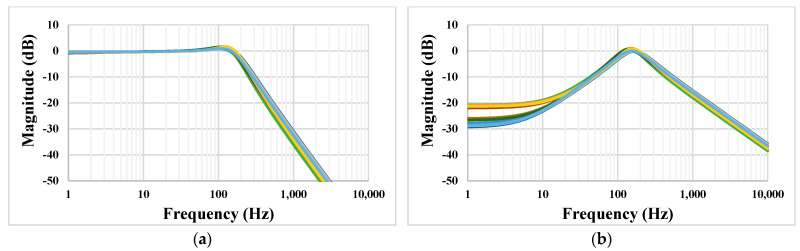
The PVT simulation of the LPF (**a**) and BPF (**b**).

Figure 12 shows the simulation results of the LPF and BPF with the process, voltage, and temperature variations. The process corners were fast-fast, fast-slow, slow-fast, and slow-slow, the voltage supply corners were in the range of V_DD_ ± 10%, and the temperature corners were 0 °C and 70 °C.

**Figure 13:**
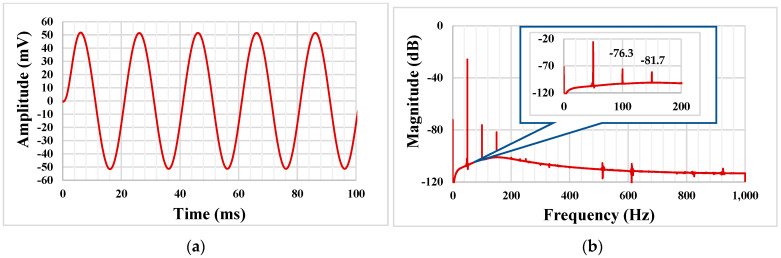
The transient response of the LPF (**a**) and its spectrum (**b**).

Figure 13 shows the transient response of the LPF with an applied input signal of 100mV_pp_ @ 50Hz and its output spectrum. The total harmonic distortion (THD) of 0.33% was achieved, which was kept still below 1% for the input signal of 200 mV_pp_ @ 50 Hz. The output integrated noise of the LPF was 220 µV_rms_ which resulted in a 50 dB dynamic range (DR = 20 × log (V_rms-max_/V_rms-onoise_)) of the filter with 1% THD.

Table 3 shows a comparison of the proposed filter with the others [26,27,28]. It is evident that the proposed filter offers the largest amount of filtering functions with a minimum count of active elements, and the lowest voltage supply, and is the only one with nanopower consumption. All these facts confirm the usability of the multiple-input *G_m_* stage in filter applications mainly by means of reducing the count of active blocks and power consumption. The figure of merit (FoM) is also presented, where a lower FoM implies the better performance of the filter. 

## 4. Conclusions

This paper demonstrates the advantages of the MI-G_m_ in filter application, in terms of topology simplification, increasing filter functions, and minimizing the count of the needed active blocks and their power consumption. Therefore, the developed circuit is a good candidate for extremely low-power low-voltage applications like biosignals processing. The filter application offers the largest amount of filtering functions with a minimum count of active elements. The post-layout simulations prove the presented advantages of MI-G_m_.

## Figures and Tables

**Table 1:** Variant filtering functions of the universal filter.

Filtering Function		Input	Output
LP	Non-inverting	$V_{in4}\;V_{in5}\;V_{in1}\;V_{in1}$	$V_{o1}\;V_{o1}\;V_{o2}\;V_{o3}$
	Inverting	$V_{in3}\;V_{in6}\;V_{in1}\;V_{in2}$	$V_{o1}\;V_{o1}\;V_{o2}\;V_{o3}$
BP	Non-inverting	$V_{in1}$ and $V_{in6}$ $V_{in3}\;V_{in6}\;V_{in3}$	$V_{o1}\;V_{o2}\;V_{o2}\;V_{o3}$
	Inverting	$V_{in2}$ and $V_{in5}$ $V_{in4}\;V_{in5}\;V_{in4}$	$V_{o1}\;V_{o2}\;V_{o2}\;V_{o3}$
HP	Non-inverting	$V_{in5}\;\mathrm{and}\;V_{in2}$	$V_{o3}$
	Inverting	$V_{in6}\;\mathrm{and}\;V_{in1}$	$V_{o2}$
BS	Non-inverting	$V_{in5}$	$V_{o3}$
	Inverting	$V_{in6}$	$V_{o3}$
AP	Non-inverting	$V_{in5}\;\mathrm{and}\;V_{in4}$	$V_{o3}$
	Inverting	$V_{in5}\;\mathrm{and}\;V_{in3}$	$V_{o3}$

Note: the unused inputs should be grounded.

**Table 2:** Transistor Aspect Ratio of the *G_m_*.

Device Name	W/L (µm⁄µm)
M_1_, M_2_, M_7_–M_10_, M_13_	2 × 15/1
M_3_–M_6_	2 × 10/1
M_3c_–M_6c_	10/1
M_7c_–M_10c_, M_13c_, M_11_, M_12_	15/1
M_L_	5/4

**Table 3:** Comparison with other filters.

	This Work	[26]	[27]	[28]
Technology (nm)	180	commercial IC	180	180
V_DD_ (V)	0.5	±15	1.2	±0.3
Power consumption (nW)	37	860 × 10^6^	0.96 × 10^6^	5770
DR (dB)	50			53.2
Fter function	22 (VM)	13 (VM)	22(VM)	20 (MM)
Offer inverting and non-inverting of five standard responses	Yes	No	Yes	No
Natural frequency (kHz)	0.153	217	1	5
Number of active and passive element	3-OTA, 2-C	5-OTA, 2-C	4-OTA, 2-C	8-OTA, 2-C
Total harmonic distortion (%)	0.33@100 mV_pp_	1.93@200 mV_pp_	1.67@600 mV_pp_	<2@200 mV_pp_
$FOM=\frac{P_{diss}}{f_{o}\times N\times DR}$	2.41 × 10^−12^	-	78.6	1.26 × 10^−12^

where $P_{diss}$ is the power dissipation, $f_{o}$ is the center frequency, N is the order of filter, and DR is the dynamic range.

## References

[1] Kwak J.Y., Park S.-Y. (2021). Compact Continuous Time Common-Mode Feedback Circuit for Low-Power, Area-Constrained Neural Recording Amplifiers. Electronics.

[2] Tasneem N.T., Mahbub I. (2021). A 2.53 NEF 8-bit 10 kS/s 0.5 μm CMOS Neural Recording Read-Out Circuit with High Linearity for Neuromodulation Implants. Electronics.

[3] Ballo A., Pennisi S., Scotti G. (2021). 0.5 V CMOS Inverter-Based Transconductance Amplifier with Quiescent Current Control. J. Low Power Electron. Appl..

[4] Nordi T.M., Gounella R.H., Luppe M., Junior J.N.S., Fonoff E.T., Colombari E., Romero M.A., Carmo J.P.P.D. (2022). Low-Noise Amplifier for Deep-Brain Stimulation (DBS). Electronics.

[5] Wyszynski A., Schaumann R. (1992). Using multiple-input transconductors to reduce number of components in OTA-C filter design. Electron. Lett..

[6] Gopinathan V., Tsividis Y.P., Tan K.-S., Hester R.K. (1990). Design considerations for high-frequency continuous-time filters and implementation of an antialiasing filter for digital video. IEEE J. Solid-State Circuits.

[7] Mourabit A.E., Lu G.-N., Pittet P. (2005). Wide-linear-range subthreshold OTA for low-power, low-Voltage, and low-frequency applications. IEEE Trans. Circuits Syst. I Regul. Pap..

[8] Munoz F., Torralba A., Carvajal R.G., Tombs J., Ramirez-Angulo J. (2001). Floating-gate-based tunable CMOS low-voltage linear transconductor and its application to HF g/sub m/-C filter design. IEEE Trans. Circuits Syst. II Analog. Digit. Signal Process..

[9] Lopez-Martin A.J., Ramirez-Angulo J., Carvajal R.G., Acosta L. (2008). CMOS transconductors with continuous tuning using FGMOS balanced output current scaling. IEEE J. Solid-State Circuits.

[10] Rodriguez-Villegas E. (2006). Low Power and Low Voltage Circuit Design with the FGMOS Transistor.

[11] Rico-Aniles H.D., Ramirez-Angulo J., Lopez-Martin A.J., Carvajal R.G. (2020). 360 nW gate-driven ultra-low voltage CMOS linear transconductor with 1 MHz bandwidth and wide input range. IEEE Trans. Circuits Syst. II Express Briefs.

[12] Khateb F., Kulej T., Kumngern M., Psychalinos C. (2019). Multiple-input bulk-driven MOS transistor for low-voltage low-frequency applications. Circuits Syst. Signal Process..

[13] Khateb F., Kulej T., Veldandi H., Jaikla W. (2019). Multiple-input bulk-driven quasi-floating-gate MOS transistor for low-voltage low-power integrated circuits. AEU-Int. J. Electron. Commun..

[14] Khateb F., Kulej T., Kumngern M., Jaikla W., Ranjan R.K. (2019). Comparative performance study of multiple-input bulk-driven and multiple-input bulk-driven quasi-floating-gate DDCCs. AEU-Int. J. Electron. Commun..

[15] Khateb F., Kulej T., Akbari M., Tang K.-T. (2022). A 0.5-V multiple-input bulk-driven OTA in 0.18-μm CMOS. IEEE Trans. Very Large Scale Integr. (VLSI) Syst..

[16] Krummenacher F., Joehl N. (1988). A 4-MHz CMOS continuous-time filter with on-chip automatic tuning. IEEE J. Solid-State Circuits.

[17] Furth P.M., Andreou A.G. (1995). Linearised differential transconductors in subthreshold CMOS. Electron. Lett..

[18] Tsividis Y.P., McAndrew C. (2010). Operation and Modeling of the MOS Transistor.

[19] Sedra A.S., Smith K.C. (1988). Microelectronic Circuit.

[20] Psychalinos C., Kasimis C., Khateb F. (2018). Multiple-input single-output universal biquad filter using single output operational transconductance amplifiers. AEU-Int. J. Electron. Commun..

[21] Jaikla W., Talabthong P., Siripongdee S., Supavarasuwat P., Suwanjan P., Chaichana A. (2019). Electronically controlled voltage mode first order multifunction filter using low-voltage low-power bulk-driven OTAs. Microelectron. J..

[22] Singh D., Paul S.K. Voltage Mode Third-Order Universal Filter Using a Single CCII. Proceedings of the 2020 7th International Conference on Signal Processing and Integrated Networks (SPIN).

[23] Unuk T., Yuce E. (2022). A mixed-mode filter with DVCCs and grounded passive components only. AEU-Int. J. Electron. Commun..

[24] Wang S.-F., Chen H.-P., Ku Y., Chen P.-Y. (2019). A CFOA-based voltage-mode multifunction biquadratic filter and a quadrature oscillator using the CFOA-based biquadratic filter. Appl. Sci..

[25] Bhaskar D.R., Raj A., Senani R. (2022). Three new CFOA-based SIMO-type universal active filter configurations with unrivalled features. AEU-Int. J. Electron. Commun..

[26] Wang S.-F., Chen H.-P., Ku Y., Yang C.-M. (2019). Independently tunable voltage-mode OTA-C biquadratic filter with five inputs and three outputs and its fully-uncoupled quadrature sinusoidal oscillator application. AEU-Int. J. Electron. Commun..

[27] Kumngern M., Khateb F., Kulej T., Psychalinos C. (2021). Multiple-input universal filter and quadrature oscillator using multiple-input operational transconductance amplifiers. IEEE Access.

[28] Namdari A., Dolatshahi M. (2022). Design of a low-voltage and low-power, reconfigurable universal OTA-C filter. Analog. Integr. Circuits Signal Process..

[29] Pevarez-Lozano H., Sanchez-Sinencio E. (1991). Minimum parasitic effects biquadratic OTA-C filter architectures. Analog. Integr. Circuits Signal Process..

[30] Sun Y., Fidler J.K. (1996). Synthesis and performance analysis of universal minimum component integrator-based IFLF OTA-grounded capacitor filter. IEE Proc. Circuits Devices Syst..

